# Acute mediastinal mass syndrome after surgical biopsy of a massive anterior mediastinal tumor: a case report

**DOI:** 10.1186/s44215-024-00131-z

**Published:** 2024-02-21

**Authors:** Masao Kobayashi, Toru Kimura, Hideki Nagata, Eriko Fukui, Takashi Kanou, Naoko Ose, Soichiro Funaki, Masako Kurashige, Eiichi Morii, Yasushi Shintani

**Affiliations:** 1 Department of General Thoracic Surgery, Osaka University Graduate School of Medicine, 2-2-L5, Yamadaoka, Suita, Osaka, 565-0871 Japan; 2 Department of Pathology, Osaka University Hospital, Suita, Osaka, Japan

**Keywords:** Mediastinal lymphatic tumor, Surgical biopsy, Mediastinal mass syndrome, Negative pressure pulmonary edema, Extracorporeal circulation, Case report

## Abstract

**Background:**

Mediastinal lymphatic tumors are relatively rare. The prognosis is poor but has improved due to recent advances in treatment strategies. Herein, we report a case of mediastinal lymphoma diagnosed using surgical biopsy, which was complicated by mediastinal mass syndrome due to general anesthesia.

**Case presentation:**

A 25-year-old man with cough, fever, dyspnea, and night sweats was transferred to our hospital for resection of a large anterior mediastinal tumor. Although his preoperative diagnosis was WHO type B1 thymoma, the clinical findings suggested a lymphoma. A repeat surgical biopsy was performed under general anesthesia. Immediately after extubation, the patient developed acute respiratory failure with hypolucency of the right lung field on chest radiography. He was reintubated immediately and was diagnosed with negative pressure pulmonary edema in the right lung. He was managed with positive-pressure ventilation and his respiratory distress resolved within 5 days. Pathological examination of surgical specimens confirmed the diagnosis of lymphoma.

**Conclusion:**

Surgical biopsy is useful for the diagnosis of mediastinal tumors. However, the risk of perioperative mediastinal mass syndrome should be carefully assessed before administering general anesthesia.

## Background

Mediastinal lymphatic tumors are relatively rare, accounting for approximately 3.2% of all mediastinal tumors [1]. Some mediastinal lymphomas have highly aggressive behavior but a relatively good prognosis [2]; hence, early and accurate diagnosis is essential for their management. Surgical biopsy is reliable for diagnosis, but general anesthesia can lead to severe cardiorespiratory problems known as mediastinal mass syndrome (MMS) [3]. We report a case of MMS in a man with a mediastinal lymphoma who underwent a diagnostic surgical biopsy under general anesthesia.

## Case presentation

A 25-year-old man presented with an initial complaint of cough, followed by spiking fever, dyspnea, and night sweats. Computed tomography (CT) revealed a 74 × 36 × 55 mm mass in the anterior mediastinum. A CT-guided biopsy performed at another hospital revealed that the tumor was a thymoma. The patient was then transferred to our hospital for surgical resection.

Upon admission, his oxygen saturation was 97% breathing room air, his body temperature was 37.0 °C, and his other vital signs were stable; however, he had severe dyspnea in the supine position, which made it difficult for him to lie down on the bed. The patient had no previous medical history of note and had smoked half a pack of cigarettes per day for 5 years.

**Fig. 1:**
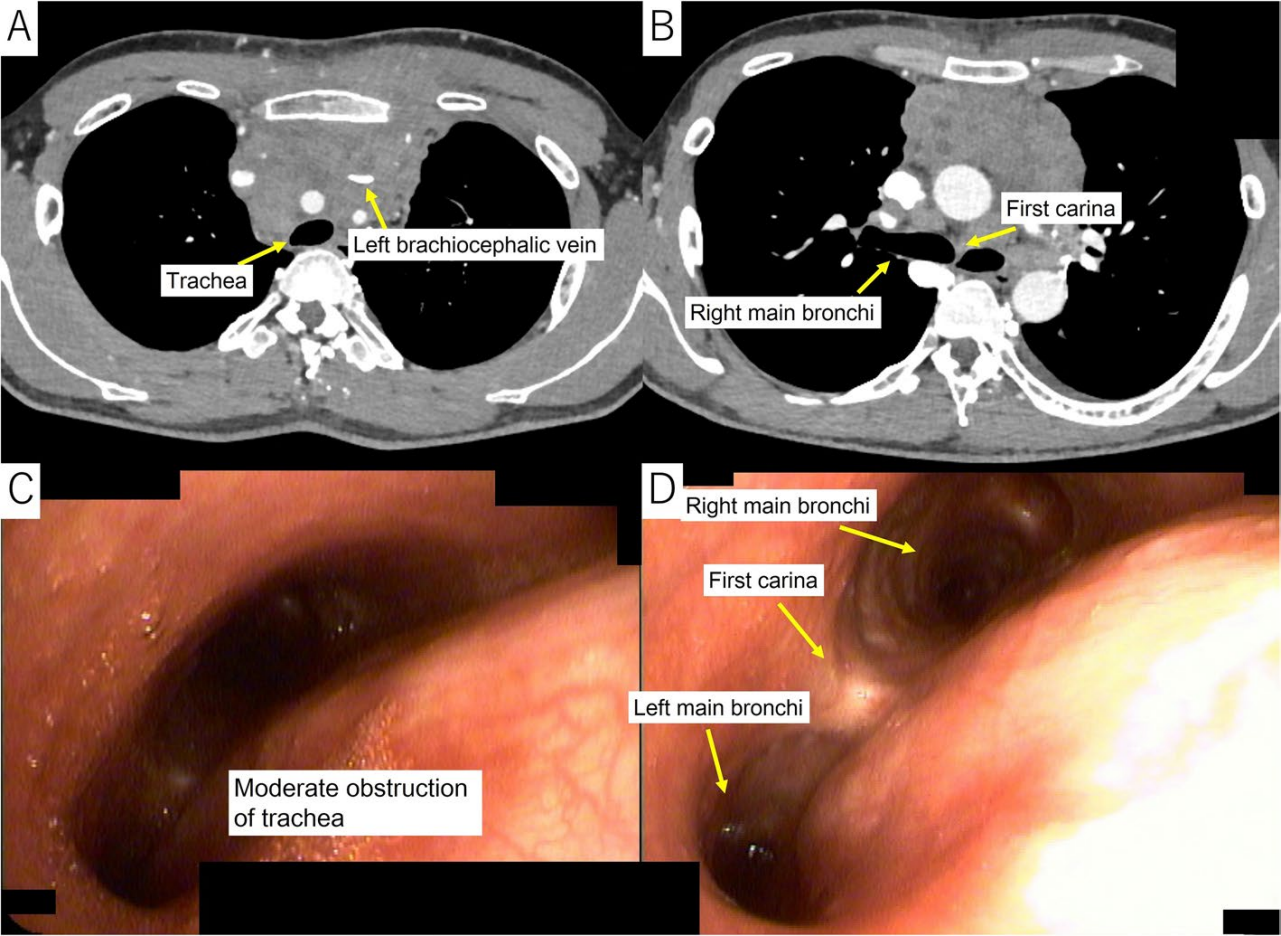
Contrast-enhanced computed tomography (CT) image showing a massive anterior mediastinal tumor with complete obstruction of the left brachiocephalic vein and moderate obstruction of the trachea (**A**), but no significant stenosis of the right main bronchus (**B**). Bronchoscopy showing moderate stenosis of the trachea (**C**) but no significant stenosis of either the left or right main bronchus (**D**), consistent with the CT images (**A** and **B**)

**Fig. 2:**
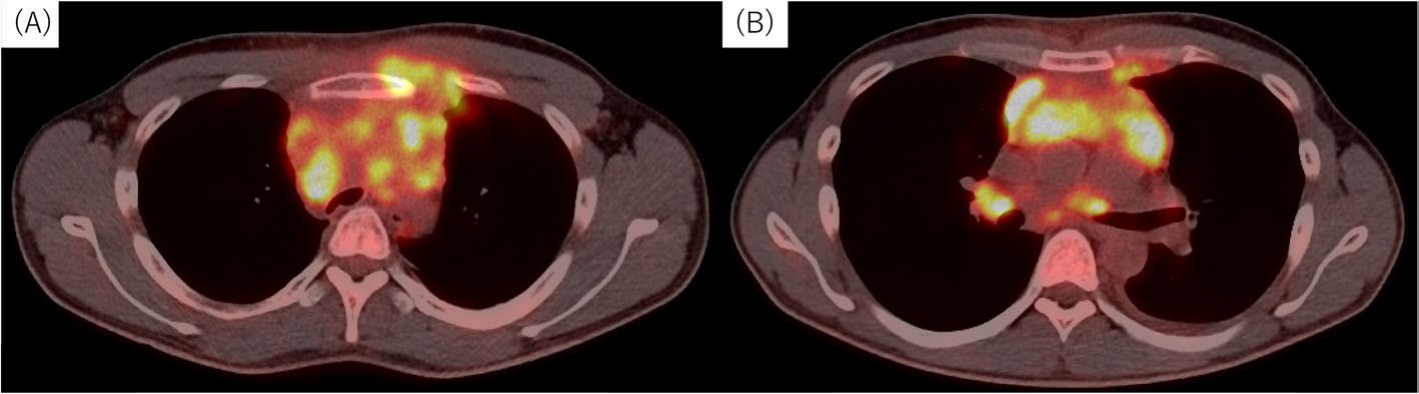
Positron emission tomography-computed tomography image showing high accumulation of the main tumor with direct invasion of the left anterior chest wall (**A**), and multiple enlarged lymph nodes (**B**)

Hematology and blood biochemistry showed an elevated white blood cell count (10,410/μL), mild anemia (serum hemoglobin 11.7 g/dL), and an elevated C-reactive protein level (11.01 mg/dL). The soluble interleukin-2 receptor level was elevated (1765 ng/mL), but the results of tests for other tumor markers were within normal limits. Chest radiography revealed a widened mediastinum. Systemic contrast-enhanced CT showed that the anterior mediastinal mass had increased in size to 75 × 40 × 100 mm, with complete obstruction of the left brachiocephalic vein and partial obstruction of the trachea (Fig. 1A), but no significant stenosis peripheral to the first carina (Fig. 1B). Bronchoscopy revealed stenosis of the trachea, consistent with the CT images (Fig. 1C and D). Positron emission tomography-CT showed high accumulation of the main tumor (maximum standardized uptake value [SUV-max]: 15.4) with direct invasion of the left anterior chest wall (Fig. 2A) and multiple enlarged lymph nodes (Fig. 2B).

**Fig. 3:**
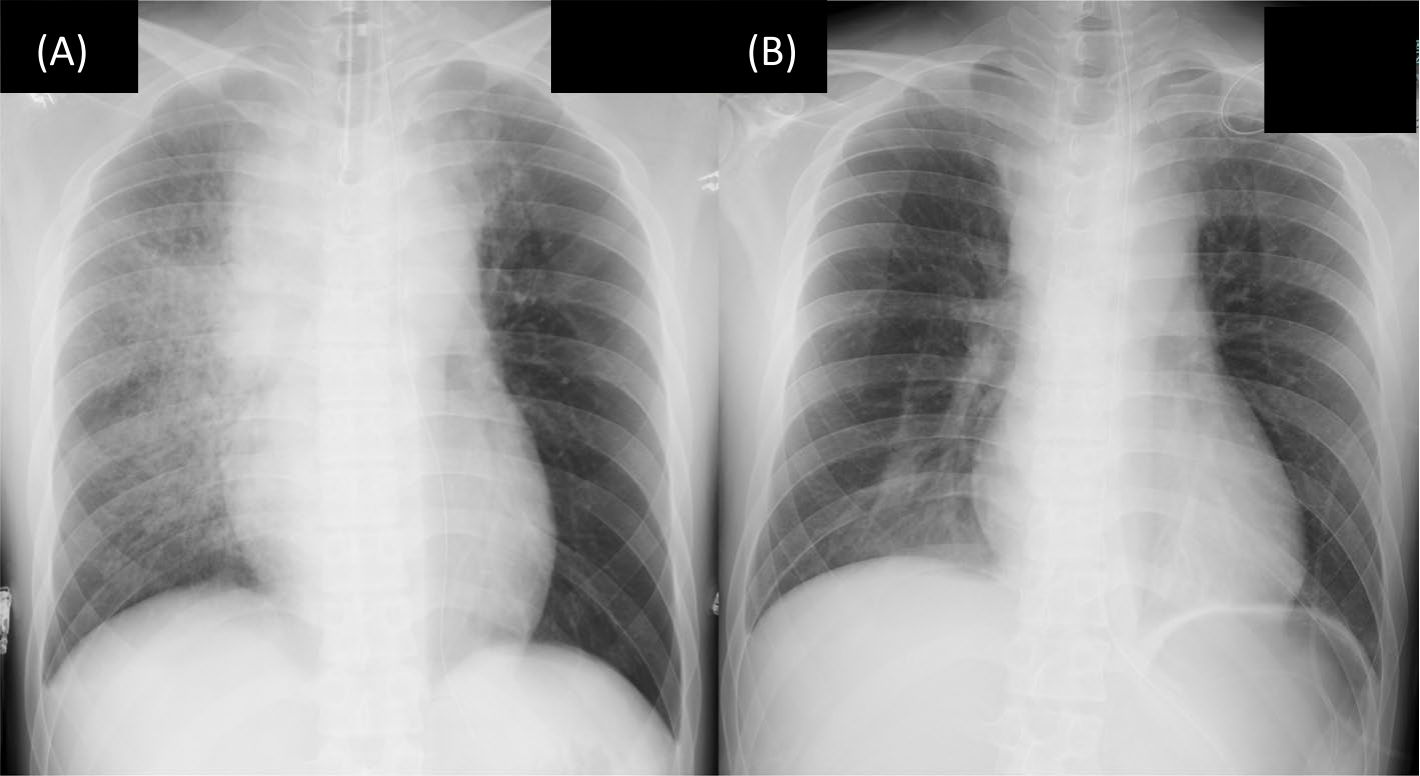
Chest radiography (**A**) showing diffuse opacities in the right lung due to negative pressure pulmonary edema after postoperative extubation in the supine position, and **B** repeat radiography on postoperative day 5 showing clearing of the opacities in the right lung after successful extubation in a semi-sitting position

Although pathological re-examination of the biopsy specimens from the previous hospital supported the diagnosis of thymoma, the history of the present illness, symptoms, laboratory data, and imaging findings suggested that the tumor was more likely to be a lymphoma than a thymoma. Owing to the discrepancy between the clinical diagnosis and the pathological diagnosis obtained from the CT-guided biopsy, surgical re-biopsy was performed. The CT findings suggested that the tracheal stenosis was not severe, so inserting an airway stent was not considered necessary. After induction of general anesthesia intubation in a semi-sitting position, biopsy specimens were percutaneously sampled from the left second intercostal space without thoracotomy. Extubation in the supine position caused negative pressure pulmonary edema (NPPE) in the right lung (Fig. 3A), which resulted in acute respiratory failure requiring immediate reintubation.

Postoperatively the patient was managed in the intensive care unit with pressure-controlled ventilation with a positive end-respiratory pressure (PEEP) of 10 cmH_2_O, without steroid therapy. He had no recurrence of respiratory decompensation and was weaned to a PEEP of 5 cmH_2_O in 4 days. He was successfully extubated in the semi-sitting position on postoperative day 5 (Fig. 3B) and was discharged on postoperative day 14.

**Fig. 4:**
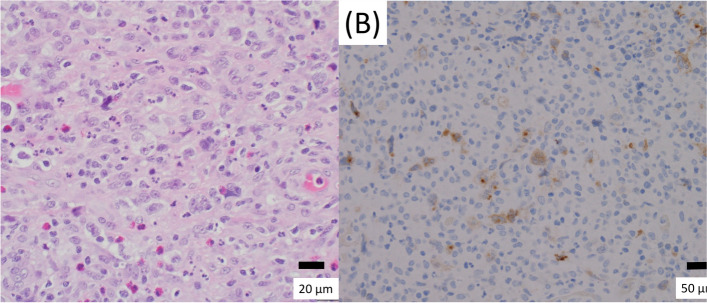
Histology of the surgical biopsy specimen showing large lymphocytes (**A**), and positive immunohistochemical staining for CD 30 (**B**)

Histological examination of the surgical biopsy specimen showed large lymphocytes (Fig. 4A) that were positive for CD 30 (Fig. 4B) on immunohistochemistry, indicating that the tumor was a lymphoma, not a thymoma. The tumor was finally diagnosed as a Hodgkin’s lymphoma based on the southern blot results, and the patient was referred to the hematology department for treatment.

## Discussion

Differential diagnosis of anterior mediastinal tumors includes malignant lymphoma, thymic epithelial tumors, and germ cell tumors [4]. Among them, thymomas and lymphomas are difficult to differentiate because they both contain abundant lymphocytes [5]. Although CT-guided biopsy is a minimally invasive and a useful diagnostic method for mediastinal tumors, an insufficient volume of biopsy specimens and contamination of thymic epithelial cells in the route of the biopsy needle can result in an incorrect diagnosis [6]. The diagnostic accuracy of CT-guided biopsy for diagnosing mediastinal lymphoma ranges from 41.9 to 89% [6], whereas surgical biopsy has a diagnostic accuracy of greater than 95% [7, 8]. Some case reports have described the difficulty of preoperative differentiation between thymomas and lymphomas [9, 10]. As the therapeutic strategies for thymoma and lymphoma are completely different, the correct pathological diagnosis of anterior mediastinal tumors is crucial for appropriate treatment.

Lymphoma affects the whole body, and patients generally present with systemic symptoms such as fever, night sweats, and weight loss [11]. In contrast, thymomas, such as other solid tumors, generally present with symptoms caused by local invasion, such as cough or chest pain [12]. In this case, in addition to systemic symptoms, the patient’s age at onset and rapid growth were incompatible with the diagnosis of thymoma. Therefore, surgical re-biopsy was performed, even though the pathologists at both hospitals had diagnosed thymoma using the specimen obtained from the CT-guided biopsy. As 22–68% of patients with mediastinal tumors undergo unnecessary thymectomy based on an incorrect diagnosis [13], the indication for surgery should be carefully considered in patients with mediastinal tumors. If the pathological diagnosis based on CT-guided biopsy specimens is inconsistent with the clinical presentation, surgical re-biopsy should be considered, as in this case.

Patients with large mediastinal masses are at risk of intraoperative MMS, leading to acute respiratory and hemodynamic decompensation [10]. Life-threatening MMS occurs due to mechanical compression of mediastinal structures, such as by NPPE, as in this case. Erdös and Tzanova [3] and Li et al. [14] reported that symptomatic patients are at a high risk of MMS during general anesthesia and that MMS can occur at any stage. Tan et al. [15] recommend that the extracorporeal circulation (ECC) technique should be prepared for patients with a high risk of MMS to support pulmonary and/or circulatory function. At our institution, we usually perform general anesthesia and surgery with ECC for symptomatic patients. In this case, the patient’s difficulty in lying supine suggested that the tumor compression was causing a narrowing of the airway, which was not apparent in CT images, indicating a high risk of MMS. The option of using an airway stent should be considered in patients with tracheal stenosis. Although there was not sufficient evidence to warrant the use of an airway stent in this case, Ost et al. [16] recommended that a stent should be used in cases with greater than 50% tracheal occlusion. Based on this recommendation, we did not consider an airway stent necessary in the present case. As intubation and ventilation were easy at the time of anesthesia induction, we decided that ECC was not needed, which resulted in life-threatening respiratory decompensation immediately after extubation in the supine position. In retrospect, biopsy under local anesthesia without muscle relaxants may have enabled MMS to be avoided in this case. In our opinion, the best anesthesia procedure for compromised respiratory patients such as the present case is local anesthesia without muscle relaxants, even when a percutaneous approach biopsy is essential. However, for sampling tumor tissue inside the thoracic cavity, general anesthesia may be unavoidable.

NPPE occurs after strong inspiratory effort against airway obstruction and can cause acute respiratory failure. Post-extubation laryngospasm is a common cause of NPPE [17], but cases of NPPE due to mediastinal lesions have also been described [18, 19]. In this case, NPPE only appeared in the right lung, suggesting that the right main bronchus was obstructed by the anterior mediastinal tumor after extubation. As in previous case reports [17, 20], the patient recovered rapidly without permanent damage. Based on our experience, when a patient has a large anterior mediastinal tumor, careful preoperative discussion should be held among surgeons and anesthesiologists, even if the planned surgery will only take a short time and is a simple procedure, and reintubation and/or ECC should be prepared for use in the event of an emergency. Regarding option of ECC device with or without circulatory support, we make decision by evaluation the risk of circulatory decompensation such as obstruction of heart or great vessels.

In conclusion, we report a case of acute MMS after surgical biopsy, which was necessary for confirming the diagnosis of a massive anterior mediastinal tumor. Although surgical biopsy can be useful for the diagnosis of mediastinal tumors, especially for differentiating between lymphoma and thymoma, the risk of perioperative MMS should be carefully assessed. The position of the body during intubation and extubation should be considered and surgeons should be prepared to administer ECC if needed.

## Data Availability

Raw data were generated at the Department of Chest Surgery at Osaka University Hospital. The derived data supporting the findings of this study are available from the corresponding author, T. Kimura, on reasonable request.

## Abbreviations

CT: Computed tomography
ECC: Extracorporeal circulation
MMS: Mediastinal mass syndrome
NPPE: Negative pressure pulmonary edema
PEEP: Positive end-respiratory pressure
SUV-max: Maximum standardized uptake value
